# Genetic diversity and antimicrobial resistance profiles of Salmonella enterica in the broiler supply chain in Harare, Zimbabwe: tracking transmission from farm to table

**DOI:** 10.1099/mgen.0.001550

**Published:** 2025-11-05

**Authors:** Peter Katsande, Alistair R. Davies, Christian Owusu-Nyantakyi, Claudious Gufe, Shuvai Musari, Chenai S. Majuru, Jairus Machakwa

**Affiliations:** 1 Department of Veterinary Technical Services, Central Veterinary Laboratories, Box CY55, 18A Liberation Legacy Way, Harare, Zimbabwe; 2 FAO Reference Centre for AMR, Department of Bacteriology, Animal and Plant Health Agency, Addlestone, UK; 3 Department of Bacteriology, Noguchi Memorial Institute for Medical Research, University of Ghana, Accra, Ghana; 4 Department of Veterinary Field Services, Bevan Building, 18A Liberation Legacy Way, Harare, Zimbabwe

**Keywords:** antimicrobial resistance, broiler supply chain, genetic diversity, *Salmonella enterica*, whole-genome sequencing

## Abstract

*Salmonella enterica* is a significant zoonotic pathogen, posing a serious public health threat through its presence in the food supply chain, particularly in poultry production facilities. This study aimed to investigate the genetic diversity, antimicrobial resistance (AMR) profiles and phylogenetic relationships of *S. enterica* serovars isolated from various stages of the broiler supply chain in Harare, Zimbabwe. Whole-genome sequencing was employed to analyse 28 *Salmonella* isolates from broiler farms, slaughter facilities and retail markets. The overall prevalence of *Salmonella* was 5.1% out of 552 samples tested. Contamination rates were higher at slaughter facilities, where 11% of 100 samples tested positive and at retail markets, where 20% of 20 samples were contaminated. In contrast, farms had a significantly lower prevalence, with only 3.0% of 432 samples showing *Salmonella* presence. Eight serovars were identified, with *S. enterica* subsp. *enterica* serovar Typhimurium being the most prevalent at 27.6%. Notably, 34.5% of the isolates harboured resistance genes, including *fosA3*, *fosA7.2* and *qnrB19*, and exhibited mutations in the *gyrA* and *parC* regions. An extended-spectrum beta-lactamase-producing *S. enterica* subsp. *enterica* serovar Kentucky ST198 strain was isolated from retail chicken cuts. All isolates carried virulence genes such as *iroC*, *iroB* and *sinH*, with *sodC1* present in 47.4% of the isolates. Approximately 31% of the isolates co-harboured antimicrobial, stress tolerance and virulence genes. Genomic analysis identified distinct sequence types while also revealing identical core SNPs in genomes across various stages of the supply chain. This study highlights the transmission of *Salmonella* and AMR in the broiler supply chain, emphasizing the urgent need for improved surveillance and intervention strategies to reduce public health risks from contaminated poultry products.

**Impact Statement**

Effective surveillance of *Salmonella* in the food supply chain is essential for informed decision-making and appropriate public health interventions aimed at improving food safety. In this study, we utilized whole-genome sequencing to provide vital insights into the genetic diversity, phylogenetic relationships and AMR genes across farms, slaughter facilities and retail markets in Zimbabwe. We subsequently identified diverse serovars including a multidrug-resistant isolate harbouring third-generation cephalosporin resistance. This study illustrates how genomics has redefined public health surveillance and contributes significantly to the literature on foodborne pathogens and zoonotic disease transmission in Zimbabwe, where such detailed genomic surveillance is limited.

**Outcome**

This study offers valuable contributions to foodborne pathogen research by utilizing whole-genome sequencing. It addresses a significant knowledge gap, particularly in regions like Zimbabwe where genomic data is scarce, highlighting the urgent need for targeted surveillance in high-risk contamination areas, such as slaughterhouses and retail markets. The findings are of broad interest to a range of stakeholders, including public health officials, food safety authorities, poultry industry professionals and researchers focusing on zoonotic diseases and antimicrobial resistance (AMR). This research is not only relevant to Zimbabwe but also to low- and middle-income countries with limited AMR monitoring systems and genomic data on foodborne pathogens. Its broader relevance supports global efforts to tackle AMR by informing both national and international food safety strategies and regulations.

**Significance as a BioResource to the community**

The study utilizes whole-genome sequencing to track *Salmonella* at different stages of the food supply chain, providing crucial data to improve food safety in Zimbabwe. The collected genome data serves as a valuable resource for understanding pathogen transmission in specific geographical contexts. It offers a framework to investigate the genetic diversity of strains among various sources and explore potential epidemiological connections between isolates throughout Zimbabwe.

## Data Summary

Sequence data reported for the first time in this study have been deposited in the National Center for Biotechnology Information Sequence Read Archive (SRA) under the BioProject PRJNA1154600, and accession numbers are listed in supplementary data (Table S1, available in the online Supplementary Material). All other sequence data used in the analysis are in available databases accessible using accession numbers. One supplementary Excel file is available with the online version of this article.

## Introduction

*Salmonella* is an important zoonotic foodborne pathogen that causes gastrointestinal infections in humans and has significant economic and public health impacts globally [1]. In the USA, *Salmonella* remains one of the most burdensome foodborne pathogens, with ~1.35 million cases of salmonellosis, 26,500 hospitalizations and 420 deaths reported each year [2]. Low- and middle-income countries experience the highest death rates, with Sub-Saharan Africa recording rates as high as 20–25% [3]. According to a review study by Ramtahal *et al*. [4], *Salmonella* was responsible for 19.7% of foodborne illnesses in South Africa. In Zimbabwe, *Salmonella* continues to be a significant foodborne pathogen and a leading cause of diarrhoeal diseases [5,6].

Poultry and poultry products are the principal sources of *Salmonella* and are potentially responsible for most foodborne zoonotic *Salmonella* transmission [7]. *Salmonella* can infect flocks in poultry farms and contaminate slaughter facilities through horizontal transmission via contaminated faeces, litter, feed, water, rodents, equipment and infected farm personnel [8]. Carcasses can be contaminated with *Salmonella* from the digestive tract during the defeathering and evisceration processes [9].

Despite numerous efforts to reduce *Salmonella* contamination in the food chain, the persistence of this pathogen in poultry products and related environments remains a global concern [10]. Compounding the problem is the worrying emergence of multidrug-resistant (MDR) *Salmonella* strains, which can spread to humans via the food pathway. Additionally, the pathogenic potential of *Salmonella* has been linked to the expression of various genes that promote host cell invasion, intracellular survival and stress tolerance in processing environments [11]. Sources and transmission routes of *Salmonella* along the food chain are not well understood, particularly in resource-limited settings due to the lack of integrated epidemiological surveillance systems. Gaining knowledge of such transmission dynamics is essential for developing effective *Salmonella* control programmes in the poultry value chain.

Diverse non-typhoidal *Salmonella* serovars, including those belonging to O-antigen groups O:4 (group B), O:7/O:8 (group C) and O:9 (group D), have been isolated from chicken farms in Zimbabwe [12]. An MDR *S*. Kentucky ST198 strain linked to both human clinical infections and poultry farms has also been reported [13]. However, *Salmonella* strains circulating within poultry production facilities have not been comprehensively investigated using a farm-to-fork approach. There is a significant knowledge gap regarding the resistance genes, virulence factors and genetic relatedness of isolates throughout the broiler supply chain. For the effective prevention of *Salmonella,* understanding the distribution of serovars along the broiler supply chain will provide valuable information regarding contamination routes and the possible persistence of serovars in the chain.

In this study, we examined the genomic diversity and phylogenetic relationships of *Salmonella enterica* strains circulating in poultry farm environments, slaughterhouses and retail chicken meat in Harare, Zimbabwe. Through whole-genome sequencing (WGS), we assessed the phylogenetic relatedness, antimicrobial resistance (AMR) profiles, virulence factors and stress tolerance of each isolate, aiming to enhance our understanding of transmission dynamics and the potential risks posed by *Salmonella* along the food supply chain.

### Study design and sample collection

A longitudinal surveillance study was conducted over a 28-month period (August 2021–December 2023) to investigate the genetic diversity and AMR profiles of *S. enterica* circulating in the broiler supply chain in and around Harare, Zimbabwe. Twelve sites were included: four broiler farms (farms A–D), two commercial slaughterhouses (SH1 and SH2) and six retail markets (R1–R6), all located within a 40-km radius of Harare, a major hub for poultry production and distribution. All four farms were integrated into the commercial supply chain and routinely delivered birds to the two participating slaughterhouses.

A total of 552 samples were collected across the broiler supply chain, comprising farms (*n*=432), slaughterhouses (*n*=100) and retail markets (*n*=20). Farm samples included fresh faeces (*n*=80), feed (*n*=60), drinking water (*n*=72), wall dust (*n*=68), drinker and feeder swabs (*n*=72) and boot swabs (*n*=80). Slaughterhouse samples consisted of caecal contents (*n*=30), swabs from defeathering and evisceration machines (*n*=40) and post-chill carcass rinsates (*n*=30). Retail market samples consisted of chilled chicken cuts (*n*=20) supplied by the two participating slaughterhouses.

### Isolation and identification

Bacterial isolation and identification were performed following the World Organisation for Animal Health Terrestrial Manual for isolation of *Salmonella* from food, feedstuffs, faecal and environmental samples [14]. Briefly, 10–25 g of samples were pre-enriched in 1 : 10 vol/vol Buffered Peptone Water (BPW) (Oxoid, Basingstoke, Hampshire, UK) and incubated for 16–20 h at 36±2 °C. Modified Semi-solid Rappaport-Vassiliadis (MSRV) agar plates (ISO) (Oxoid) and Müller–Kauffmann Tetrathionate broth (Oxoid) were inoculated with 0.1 ml and 1 ml of incubated BPW, respectively. The MSRV plates were incubated at 41.5±1 °C, while Tetrathionate broth was incubated at 36±2 °C. After 24 and 48 h of selective enrichment, 1 µl loop of material from the edge of the turbid growth zone on MSRV and 10 µl of Tetrathionate broth were streaked onto xylose lysine deoxycholate agar (XLD) (Oxoid) and Brilliance™ *Salmonella* Agar (Oxoid). The plates were incubated at 36±2 °C for 21–27 h. Plates were examined for presumptive *Salmonella* colonies, which appeared on XLD as opaque/yellow, pink or red colonies with or without black centres and on Brilliance *Salmonella* Agar as purple colonies. Presumptive *Salmonella* colonies were purified on nutrient agar (Oxoid), and species identification was confirmed by the API-20E biochemical test (API-20, BioMerieux, UK and Ireland) and slide agglutination with polyvalent ‘O’ (A-S) and poly ‘H’ (phase 1 and phase 2 antisera) (Remel). *S*. Typhimurium ATCC 14028 and *Escherichia coli* ATCC 25922 were used as positive and negative control strains, respectively.

### WGS and analysis

The *Salmonella* isolates were sub-cultured on nutrient agar and incubated overnight at 37 °C. Genomic DNA was extracted using QIAamp DNA mini kits (Qiagen) as per the manufacturer’s instructions. DNA libraries were prepared using the Nextera XT (Illumina) sample preparation kit. The isolates were then sequenced on an Illumina NextSeq (San Diego, CA, USA) platform, generating 150 bp paired-end reads. FastQC v0.11.9 [15] and MultiQC v1.11 [16] were utilized to evaluate the quality of reads. PrinSEQ v0.20.4 [17] was employed to clean, filter and trim the raw reads, removing those with a mean quality score below 28 and trimming the first 10 bases from the left end of the reads. Draft genome assemblies were generated using SPAdes v3.14.1 at default settings [18]. The quality of these assemblies was evaluated with QUAST v5.0.2 [19]. The presence of genes and point mutations conferring AMR, virulence and heavy metal stress tolerance was assessed using ResFinder v4.5.0 [20] and AMRFinderPlus v3.12.8 [21]. The sequence type (ST) was determined with MLST v2.19.0 [22], using the pubMLST database [23]. *In silico Salmonella* serotyping was carried out using the SISTR command line tool [24]. Core genome SNPs were generated using SNIPPY [25], using *S. enterica* subsp. *enterica* serovar Typhimurium str. LT2 (accession: NC_003197) as a reference strain. Phylogenetic trees with 200 bootstraps were built using RAX-ML [26] from the core genome SNPs and annotated using iTOL v5 [27]. Sequence data is available in the National Center for Biotechnology Information Sequence Read Archive (SRA) under the BioProject PRJNA1154600.

SNP analysis was performed to compare *S. enterica* serovar Kentucky ST198 strains isolated from retail chicken cuts with human clinical strains obtained in Zimbabwe, specifically from Harare (*n*=6), Chitungwiza (*n*=1), Mutare (*n*=1) and Kadoma (*n*=1) [13]. The raw sequencing reads for the human clinical isolates are publicly available in the Sequence Read Archive (SRA) under study accession number PRJNA762287 (https://www.ncbi.nlm.nih.gov/sra).

## Results

### Prevalence and distribution of *Salmonella* in the broiler supply chain

**Fig. 1.**
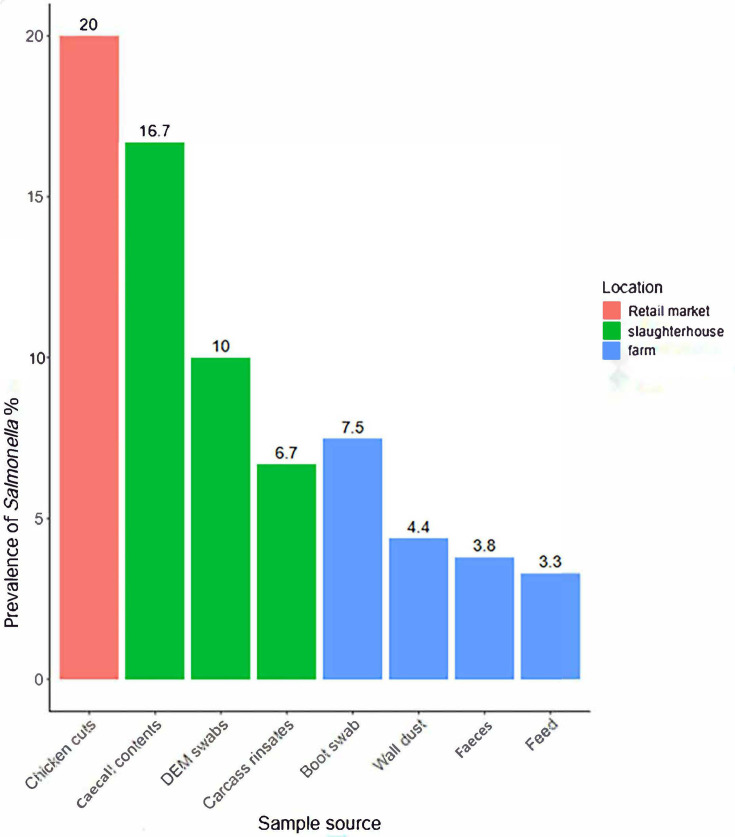
Prevalence of *Salmonella* in broiler farms, slaughterhouses and retail markets.

The overall prevalence of *Salmonella* in the broiler supply chain was 5.1%(28/552). Retail markets had the highest contamination rate at 20.0% (4/20), followed by slaughterhouses at 11.0% (11/100) and farms at 3.0% (13/432) (Fig. 1).

All *Salmonella* isolates from the retail market originated exclusively from chicken cuts. In slaughterhouses, the highest *Salmonella* prevalence was detected in caecal contents at 16.7% [95% confidence interval (CI): 5.6–34.7%), followed by swabs from defeathering and evisceration machines at 10.0% (95% CI: 2.8–23.7%) and carcass rinsates at 6.7% (95% CI: 0.8–22.1%). On farms, *Salmonella* was detected in boot swabs at 7.5% (95% CI: 3.1–14.9), wall dust at 4.4% (95% CI: 0.9–12.4), faeces at 3.8% (95% CI: 0.8–10.6) and feed at 3.3% (95% CI: 0.4–11.5). However, *Salmonella* was not isolated from drinking water or swabs from drinkers and feeders.

### Characterization and prevalence of *S. enterica* serovars in the broiler supply chain

**Fig. 2.**
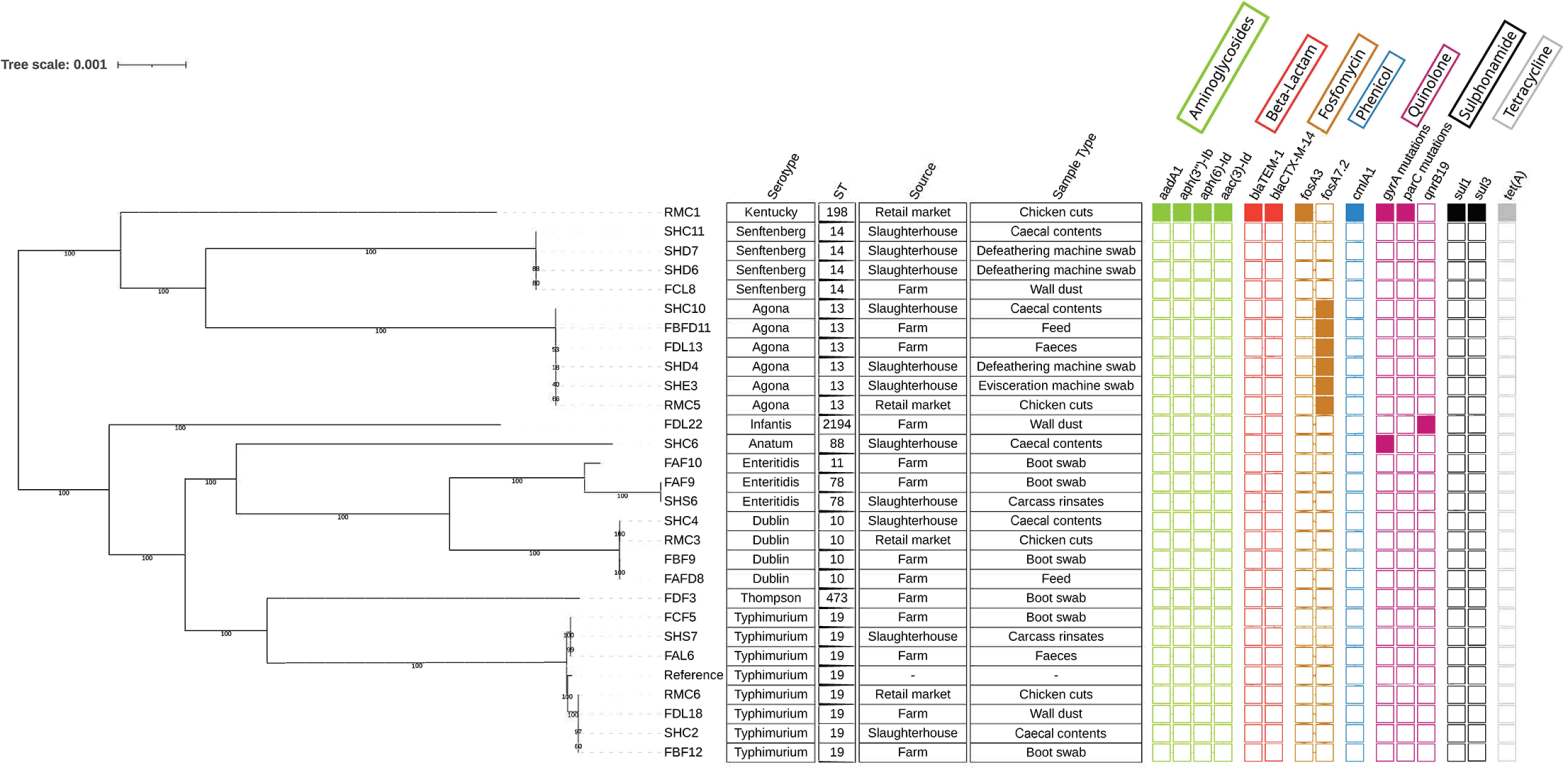
Phylogenetic tree of *Salmonella* strains identified with AMR genes. Shaded boxes indicate the presence of the AMR gene, and unshaded boxes indicate the absence of the gene. *S*. Typhimurium LT2 was used as the reference strain as it was the most prevalent serovar in this study.

All *S. enterica* isolates were identified as eight distinct serovars. The most prevalent serovar identified *in silico* was *S*. Typhimurium (*n*=7), all of which were ST19, followed by *S*. Agona (*n*=6), all ST13; *S*. Senftenberg (*n*=4), all ST14; *S*. Dublin (*n*=4), all ST10; and *S*. Enteritidis (*n*=3), with ST11 (*n*=1) and ST78 (*n*=2). *S*. Infantis (*n*=1) was identified as ST2194. The single strains of *S*. Anatum, *S*. Thompson and *S*. Kentucky were classified as ST88, ST473 and ST198 STs, respectively. Most STs were identified at multiple different sampling and production stages. Specifically, *S*. Typhimurium ST19 (isolates: FAL6, SHC2 and RMC6), *S*. Agona ST13 (isolates: FBFD11, SHC10 and RMC5) and *S*. Dublin ST10 (isolates: FAFD8, SHC4 and RMC3) were isolated from farms, slaughterhouses and retail markets. *S*. Senftenberg ST14 (isolates: FCL8 and SHC11) was isolated from both farms and slaughterhouses (Fig. 2).

### Analysis of AMR, virulence, stress tolerance and biocide resistance genes

A total of 15 AMR genes were detected across the broiler production chain, shared among 9 out of 28 *Salmonella* isolates (Fig. 2). The fosfomycin glutathione transferase gene (*fosA7.2*) was identified in six *Salmonella Agona* ST13 strains, isolated from different stages of the broiler production chain (farms and slaughterhouses). *fosA7.2* is regarded as a potentially silent resistance gene [28]. While it may not confer detectable resistance under normal conditions, it can become clinically significant under selective pressure or through regulatory mutations and horizontal transfer. This is supported by the elevated fosfomycin resistance MIC values (>512 mg ml^−1^) reported in previous studies [29]. One *S*. Infantis strain (FDL22) carried the pentapeptide repeat protein gene (*qnrB19*), conferring resistance to fluoroquinolone antibiotics [13]. The only *S*. Anatum strain isolated along the production chain (caecal contents) possessed a *gyrA* mutation. Notably, antibiotic resistance genes *qnrB19* and *fosA7.2* appeared to be serotype-specific as qnrB19 was found only in *S*. Infantis and *fosA7.2* was found only in *S*. Agona.

**Fig. 3.**
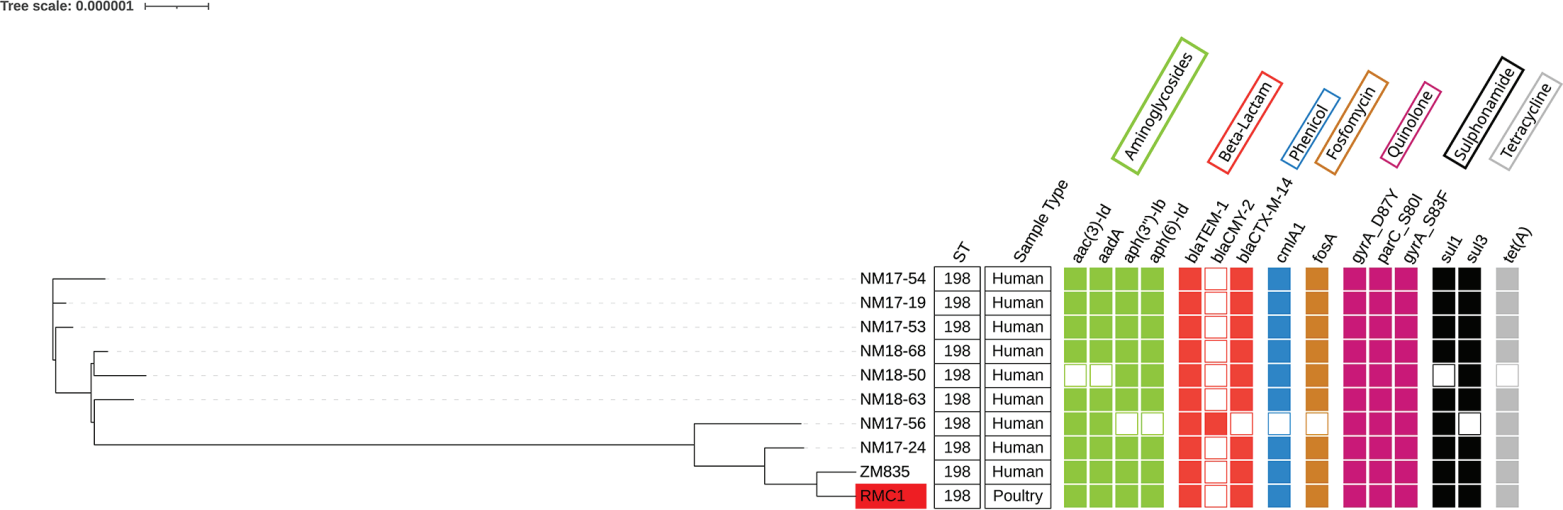
Phylogenetic tree of *S*. Kentucky ST198 strains from human clinical cases and poultry from this study. Shaded boxes indicate the presence of the AMR gene, and unshaded boxes indicate the absence of the gene.

One isolate, the *S*. Kentucky ST198 strain (RMC1) recovered from a retail market, carried multiple AMR genes conferring resistance to aminoglycosides (*aadA1*, *aph(3'')-Ib*, *aph(6)-Id* and *aac(3)-Id*), fosfomycin (*fosA3*), folate pathway antagonists (*sul1* and *sul3*), tetracycline (*tetA*) and amphenicol (*cmlA1*). Notably, this *S*. Kentucky ST198 strain also harboured genes conferring resistance to AmpC beta-lactamase (*blaCMY-2*), class A *β*-lactamase (*blaTEM-1B*) and extended spectrum *β*-lactamase (*blaCTX-M-14*), along with chromosomal point mutations in DNA gyrase (*gyrA*) and DNA topoisomerase (*parC*). This strain also shared similar antibiotic resistance profiles with *S*. Kentucky ST198 strains isolated from human clinical cases in Zimbabwe (Fig. 3).

**Fig. 4.**
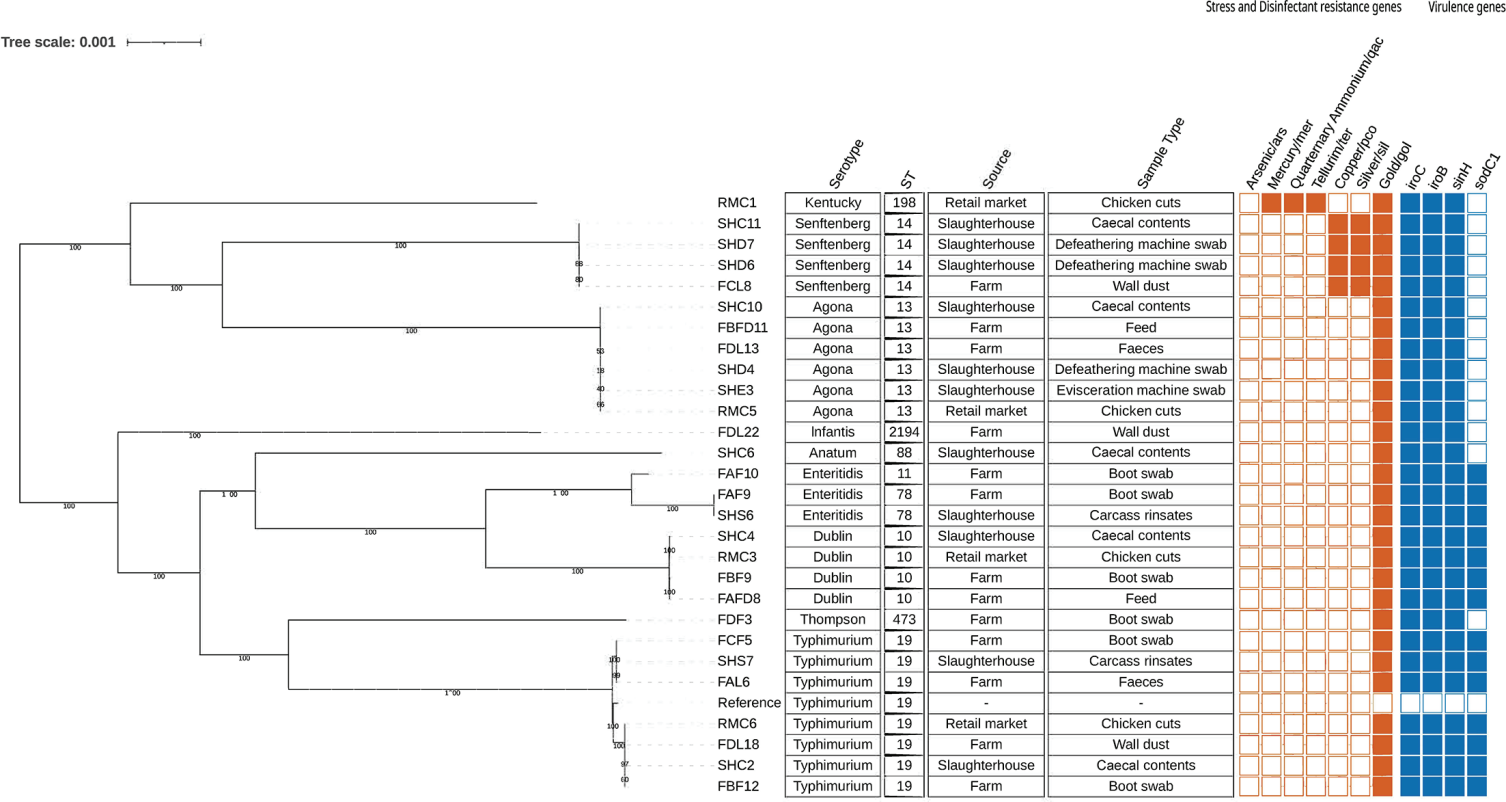
Phylogenetic tree of *Salmonella* strains identified in this study with virulence, stress response and biocide resistance genes. Shaded boxes indicate the presence of the resistance genes, and unshaded boxes indicate the absence of the gene. *S*. Typhimurium LT2 was used as the reference strain as it was the most prevalent serovar in this study.

All isolates, except for one (FCL 16), harboured the virulence genes *sinH*, *iroC* and *iroB*, which enhance the pathogenicity of *Salmonella* by promoting host cell invasion and facilitating iron uptake [30] (Fig. 4). The *sodC1* gene was found in all *S*. Enteritidis, *S*. Dublin and *S*. Typhimurium serovars. Various stress response genes were identified across different serovars. All isolates contained the gold operon (*golS* and *golT*). Interestingly, only *S*. Senftenberg serovars possessed both copper and arsenic stress genes. The *S*. Kentucky isolate carried additional mercury operon genes (*merP*, *merR*, *merT*, *merD*, *merA*, *merE* and *merC*), as well as genes conferring resistance to quaternary ammonium compounds (*qacC/qacEDelta1*) and tellurium stress response (*terD*, *terW* and *terZ*).

### Co-occurrence of antimicrobial, heavy metal and biocide resistance genes in *Salmonella* isolates from the broiler production chain

Of the 15 AMR genes, co-presence with heavy metal and biocide resistance genes was observed in 34.5% (10/29) of *Salmonella* isolates (Fig. 4). The most common co-presence was for gold and fosfomycin resistance (6/29, 20.7%), followed by gold, quinolone and fluoroquinolone resistance (2/29, 6.9%). Notably, a single *S*. Kentucky ST198 strain co-harboured genes for resistance to gold, copper, silver, arsenic, mercury and tellurite, along with resistance to aminoglycosides, beta-lactams, phosphonic, phenicol, quinolones, sulphonamides and tetracycline.

### SNP analysis among *Salmonella* isolates from the broiler production chain

Pairwise SNP comparisons among different *Salmonella* STs revealed low SNP distances within certain strains (Table S2). Specifically, *S*. Agona ST13 and *S*. Senftenberg ST14 exhibited mean SNP distances of ~6 and 10, respectively, suggesting a recent common ancestor. Despite the genetic similarity, these isolates originated from different stages of the broiler production chain. For instance, the *S*. Agona ST13 strain comprised six isolates from various sources: farm (*n*=3), slaughterhouse (*n*=2) and retail market chicken (*n*=1). Within this group, the farm isolate FBFD11 differed by 5 SNPs from the slaughterhouse isolate SHE3 and the retail market isolate RMC5, which were obtained from an evisceration machine swab and chicken cuts, respectively. Similarly, the *S*. Senftenberg isolate SHD7 from a defeathering machine swab at a slaughterhouse differed by 6 SNPs from the farm isolate FCL8, which was sourced from wall dust. Although *S*. Typhimurium ST19 displayed a higher mean SNP distance of ~529 SNPs, significant genetic similarity was still observed within this ST. Notably, the farm isolate FBF12 from a boot swab was identical (0 SNPs) to the slaughterhouse isolate SHC2 from caecal contents and differed by only 1 SNP from the retail market isolate RMC6, which was obtained from chicken cuts.

### SNP analysis among *S*. Kentucky ST198 strains from human clinical samples and retail chicken cuts from Zimbabwe

The *S*. Kentucky ST198 strain (RMC1) isolated from chicken cuts showed a mean distance of ~19 SNPs when compared to other *S*. Kentucky ST198 strains isolated from human clinical infections in Zimbabwe (Table S3). Notably, the human clinical isolates NM17-24 and ZM835 differed by only 6 and 10 SNPs, respectively, from the chicken isolate RMC1.

## Discussion

*Salmonella* contamination in poultry and poultry products can occur at various stages of the food supply chain. An analysis of *Salmonella* prevalence across the broiler production chain in Zimbabwe revealed varying contamination rates at different stages. The overall prevalence of 5.1% was lower than the 10% prevalence reported in a previous study conducted in Zimbabwe by Makaya *et al*. [12]. These discrepancies may be due to differences in geographical locations, sample sizes, farm management practices, sampling procedures and the types of samples analysed [31]. Furthermore, the presence of *Salmonella* at various stages from farms to retail markets highlights the risk of contamination along the supply chain. Similar studies have shown that *Salmonella* strains frequently spread throughout the broiler chicken supply chain [32]. As noted by Waghamare *et al*. [33], this study confirms the higher prevalence of *Salmonella* spp. from processing facilities compared to production farms. While elevated *Salmonella* isolation rates in slaughter facilities are likely attributable to contamination within the processing environment, further compounded by persistent cleaning challenges and inadequate disinfection protocols [34,35], positive samples were detected across multiple time points, from February 2023 to December 2023. These detections occurred intermittently and included isolates from both chicken cuts and slaughterhouse environments, indicating ongoing contamination rather than a single outbreak event. Notably, the highest frequency of *Salmonella* occurrence was recorded at the retail stage, which may reflect additional opportunities for cross-contamination during product handling, packaging and distribution [36]. Hygienic practices and cleanliness in these shops are often minimal, and they frequently receive potentially infected live birds from various broiler suppliers [37].

While US regulations allow for the use of antimicrobials such as peracetic acid in immersion chilling [38], this practice is not typical in Zimbabwe, where slaughterhouses rely solely on plain water chilling without chemical decontaminants. This lack of intervention, combined with variable hygienic standards and limited temperature control, likely facilitates *Salmonella* persistence on carcasses post-processing. At the farm level, biosecurity is also inconsistently applied, particularly in small- to medium-scale operations that dominate local production. Mixed farming systems are common, with poultry raised in proximity to cattle, goats and other livestock, often under minimal biosecurity. Such conditions create opportunities for cross-species transmission, as illustrated by the unusual detection of *S*. Dublin, a cattle-adapted serovar, in poultry samples. Environmental contamination through shared equipment, water sources or personnel movement is a plausible route for its introduction. Similarly, the occurrence of *S. Agona*, though not as rare as *S. Dublin*, is also atypical in poultry and may reflect environmental persistence rather than routine host association. These findings underscore the complex ecology of *Salmonella* in Zimbabwean poultry systems, where weak biosecurity, limited hygiene and absence of national control programmes contribute to the circulation of both typical and atypical serovars.

The predominance of *S*. Typhimurium and *S*. Enteritidis in this study aligns with previous reports from Zimbabwe [12,39, 40]. However, the detection of serovars such as *S*. Agona ST13, *S*. Senftenberg ST14, *S*. Dublin ST10, *S*. Infantis ST2194, *S*. Anatum ST88 and *S*. Thompson ST473 represents either a genuine shift in *Salmonella* diversity or an improvement in detection methods. Regardless, their presence in the poultry chain is noteworthy, as these serovars are linked to human foodborne illness across sub-Saharan Africa [41,42].

WGS provided further insight into the relatedness of isolates across the production chain. The low SNP distances (<10 SNPs) among *S*. Agona ST13 and *S*. Senftenberg ST14 indicate that these strains belong to the same genetic cluster and likely share a recent common ancestor [43]. For example, the farm isolate FBFD11 showed only 5 SNP differences compared to isolates from the slaughterhouse (SHE3) and retail (RMC5), strongly suggesting clonal dissemination across the chain [44]. Likewise, *S*. Senftenberg ST14 isolates from farm wall dust (FCL8) and a slaughterhouse defeathering machine (SHD7) differed by only 6 SNPs, indicating persistence within production environments. Importantly, isolates within the same cluster shared identical gene profiles, further supporting common origins. For instance, the farm isolate FBF12, slaughterhouse isolate SHC2 and retail isolate RMC6 were genetically indistinguishable, demonstrating strain transmission from farm through slaughter to retail.

The analysis of virulence and stress response genes among *Salmonella* isolates from the poultry supply chain highlights significant adaptability and potential public health risks. In Zimbabwe, the prevalence of virulence genes in *Salmonella* from poultry was previously unknown, making this study the first to investigate *Salmonella* virulence in poultry isolates. The universal presence of virulence genes such as *sinH*, *iroC* and *iroB* underscores their critical role in *Salmonella* pathogenicity [45]. The *iroB* and *iroC* genes facilitate iron uptake from the host [46], while the *sinH* gene encodes proteins involved in cell adhesion and invasion [47]. The consistent detection of the *sodC1* gene in key serovars highlights a common strategy for evading host defences [48]. These findings suggest that healthy poultry and their environment serve as reservoirs for pathogenic *Salmonella* strains that could be capable of causing infections in humans.

Stress response genes were widespread, with all isolates carrying the gold operon. The co-occurrence of AMR genes with stress response genes in all isolates is particularly alarming, as it suggests strong selective pressures that favour the emergence of multi-resistant strains. The presence of stress response genes in *Salmonella* poses significant public health risks by enhancing pathogen resilience, potentially leading to co-resistance with antibiotics, complicating treatment options and increasing environmental persistence and virulence [49].

The presence of an extended-spectrum beta-lactamase- and AmpC-producing *S*. Kentucky ST198 isolate in chicken cuts is particularly concerning. Though detected only once, this MDR strain has been associated with high-level fluoroquinolone resistance and widespread dissemination in Africa and Europe [50,52]. The genetic similarity observed between poultry-derived *S*. Kentucky ST198 (RMC1) and human clinical isolates, with differences as low as 6–10 SNPs, suggests possible zoonotic transmission and a shared evolutionary lineage. This finding mirrors global reports identifying poultry as a reservoir for *S*. Kentucky ST198 in human infections [51].

Together, these results reinforce the interconnectedness of farms, processing plants and retail outlets as part of a continuous transmission network. The combination of weak biosecurity, lack of antimicrobial interventions, poor hygiene in slaughterhouses and informal retail practices creates an environment conducive to the persistence and spread of *Salmonella*, including MDR strains. Given these risks, enhanced biosecurity measures, stricter hygiene protocols in processing facilities and robust surveillance systems are essential to minimizing *Salmonella* transmission from poultry to humans [53]. While limited sample size and lack of longitudinal data prevent definitive conclusions, the evidence highlights a pressing need for improved biosecurity, enhanced surveillance and targeted interventions across Zimbabwe’s poultry production system. Framing these findings within a One Health perspective emphasizes that controlling *Salmonella* in poultry is essential not only for food safety but also for mitigating human health risks in Zimbabwe.

The genetic similarity between the isolates highlights the risk of cross-contamination or direct exposure to contaminated poultry products as potential transmission pathways for *Salmonella* Kentucky ST198. Given these risks, enhanced biosecurity measures, stricter hygiene protocols in processing facilities and robust surveillance systems are essential to minimizing *Salmonella* transmission from poultry to humans [53].

## Conclusion

This study reveals the dynamic nature of *Salmonella* along the broiler supply chain in Zimbabwe, characterized by varying prevalence rates and diverse genetic profiles among isolates. The findings underscore the coexistence of AMR with heavy metal and biocide resistance, indicating potential co-selection pressures that contribute to the persistence of *Salmonella*. Understanding these dynamics is essential for developing effective strategies to mitigate *Salmonella* contamination and in poultry production systems. By addressing these issues through improved surveillance, responsible antimicrobial use and enhanced biosecurity measures, we can strengthen food safety, protect public health and promote sustainable poultry production systems.

## Supplementary material

10.1099/mgen.0.001550 Uncited Table S1.

10.1099/mgen.0.001550 Uncited Table S2.

10.1099/mgen.0.001550 Uncited Table S3.

## Abbreviations

AMR: antimicrobial resistance
BPW: Buffered Peptone Water
CI: confidence interval
MDR: multidrug-resistant
MIC: Minimum inhibitory concentration
MSRV: Modified Semi-solid Rappaport-Vassiliadis
SNP: Single Nuceotide Polymorphism
SRA: Sequence Read Archive
ST: sequence type
WGS: whole-genome sequencing
XLD: xylose lysine deoxycholate agar
